# Sarcopenia and low muscle radiodensity associate with impaired FEV_1_ in allogeneic haematopoietic stem cell transplant recipients

**DOI:** 10.1002/jcsm.12604

**Published:** 2020-07-29

**Authors:** Asmita Mishra, Kevin D. Bigam, Martine Extermann, Rawan Faramand, Kerry Thomas, Joseph A. Pidala, Vickie E. Baracos

**Affiliations:** ^1^ Blood and Marrow Transplantation H. Lee Moffitt Cancer Center and Research Institute Tampa FL USA; ^2^ Department of Oncology University of Alberta Edmonton Alberta Canada; ^3^ Senior Adult Oncology Program H. Lee Moffitt Cancer Center and Research Institute Tampa FL USA; ^4^ Diagnostic Imaging and Interventional Radiology H. Lee Moffitt Cancer Center and Research Institute Tampa FL USA

**Keywords:** Sarcopenia, Allogeneic haematopoietic transplantation, Muscle radiodensity, Forced expiratory volume

## Abstract

**Background:**

Quantification of skeletal muscle using computed tomography (CT) is accessible using cancer patients' standard oncologic images. Reduced muscle mass may be related to reduced respiratory muscle strength; however, the impact of this on lung functional parameters is not characterized in adult allogeneic haematopoietic stem cell transplant (alloHCT) recipients.

**Methods:**

A consecutive retrospective series (*n* = 296) of patients who had alloHCT at a comprehensive cancer centre between March 2005 and April 2015 were included. Pre‐transplant CT scans were used to quantify skeletal muscle and adipose tissue at the fourth thoracic (T4) and/or third lumbar (L3) level. Tumour and patient characteristics were recorded, including forced expiratory volume in 1 second (FEV_1_) by spirometry. Regression models were created to characterize predictive relationships.

**Results:**

A total of 296 patients (♂*n* = 161; ♀*n* = 135) were included, all of whom had chest CT as part of standard care; a subset of these (*n* = 215, 72.6%) also had abdominal CT. Diagnoses were non‐Hodgkins lymphoma (*n* = 165), acute myeloid leukaemia (*n* = 66), Hodgkin's disease (*n* = 14), acute lymphocytic leukaemia (*n* = 14), myelodysplastic syndromes (*n* = 18), and other (*n* = 19). In multivariable linear regression adjusted for sex (*P* < 0.0001), age (*P* < 0.0001), haematopoietic cell transplantation‐specific co‐morbidity index (*P* = 0.010), and parameters of pulmonary function testing (defined by spirometry, *P* < 0.0001), both T4 muscle index [*β* 0.127 (95% confidence interval 0.019; 0.252), *P* < 0.0001] and T4 muscle radiodensity [*β* 0.132 (95% confidence interval 0.087; 0.505), *P* = 0.006] were independently associated with FEV_1_; disease risk index (*P* = 0.877) and Karnofsky performance status (*P* = 0.548) were not associated with FEV_1_. Similar conclusions were obtained when L3 muscle index and radiodensity were considered. Unlike T4, L3 muscle index values can be compared with published cut‐off values for sarcopenia. Overall rates of sarcopenia were uniformly higher in the HCT population than in age‐matched and sex‐matched patients with solid tumours [alloHCT ♂64.7% vs. solid tumour ♂56.6% (*P* < 0.001); alloHCT ♀57.6% vs. solid tumour ♀36.0% (*P* < 0.001)]. Significant but moderate correlations (*P* < 0.001) were found for muscle area and radiodensity between L3 and T4, for both men and women; adipose tissue quantity also correlated significantly (*P* < 0.001) between L3 and T4 for both men and women.

**Conclusions:**

Lumbar or thoracic CT images are useful for body composition assessment in this population and reveal high rates of sarcopenia, similar to those reported in very elderly patients. Reduced muscle mass and radiodensity associate with impaired FEV_1_ even after adjustment for clinical covariables including co‐morbidities, performance status, disease risk, and mild intrinsic pulmonary disease (chronic obstructive pulmonary disease) defined by spirometry.

## Introduction

Healthy haematopoietic stem cell transplantation from an allogeneic donor (alloHCT) is a treatment option to improve outcomes for otherwise incurable diseases. With the development of safer transplant conditioning regimens and supportive care measures, transplant can now be offered as a potential curative option to a wider population that include older, frail patients with co‐morbidities. However, the potential benefits of alloHCT can be offset by potential treatment‐related mortality. Candidate selection includes evaluation of a patient's diagnosis, stage, and remission status, but additionally patient tolerability of the procedure is influenced by co‐morbidity burden, performance status, and chronological age.^1^ Given the limitations of currently utilized performance status measures and co‐morbidity indices, additional efforts to find alternative supplemental tools have been made in hopes to better ascertain functional performance status pre‐transplantation.^2^, ^3^

Sarcopenia, severe depletion of muscle mass [indicated by low skeletal muscle index (SMI)], is characterized by impairments in strength, functional limitations, and physical disability.^4^, ^5^, ^6^ This can be, and is, readily identified via radiologic imaging in cancer patients, including those with haematologic malignancies, and is further associated with poor clinical outcomes.^7^, ^8^, ^9^ Reduced skeletal muscle radiodensity (SMR) is distinct characteristic of muscle, indicative of fatty infiltration, and this has also been related to a growing number of adverse clinical outcomes in recent years.^10^, ^11^, ^12^, ^13^ SMI and SMR have been related to physical functioning, but this has not usually included pulmonary function(s). Pulmonary complications and dysfunction after alloHCT have been described to a significant extent over the years as being a primary cause of early morbidity and mortality, particularly for their association to graft‐vs.‐host disease.^14^, ^15^, ^16^, ^17^ Thus, it is common practice to carry out functional pulmonary assessment prior to and after HCT, by measuring pulmonary function including forced expiratory volume in 1 second (FEV_1_). Many muscles are recruited for forceful expiration including, primarily, the internal intercostals, *intercostalis intimi*, and subcostals, while accessory expiratory muscles include the *rectus abdominis*, external oblique, internal oblique, and *transversus abdominis*. This degree of involvement would suggest that persons affected by substantial overall muscle wasting may have impaired forced expiration.

While the research on each of these three parameters (SMI, SMR, and FEV_1_) independently is extensive, until this point, literature that aims to associate FEV_1_ to SMI/SMR in cancer patients has been limited and absent in alloHCT. In hepatocellular carcinoma, a significant relationship between preoperative FEV_1_ and psoas muscle index was found, although the use of only the psoas muscle limits the strength of this postulation as it is not involved in respiration.^18^ Outside of cancer, multiple studies of chronic obstructive pulmonary disease (COPD) have posited a relationship between fat‐free mass index and intercostal muscle cross‐sectional area with FEV_1_ and COPD severity; however, the body composition of these patients compared with those of alloHCT likely differs significantly.^19^, ^20^ Lastly, in a large‐scale study of healthy elderly adults, regression analysis showed that computed tomography (CT)‐defined thigh muscle area was indeed an independent predictor of FEV_1_.^21^ In sum, recent publications have certainly suggested a potential relationship between body composition and FEV1, though until this point, not in HCT patients.

We hypothesized that recipients of alloHCT were likely to have sarcopenia given that those patients are often heavily pretreated with chemotherapy prior to the procedure. Thus, we aimed to evaluate patients about to receive alloHCT to determine the incidence of sarcopenia in this population. Further, we aimed to characterize how sarcopenia, as well as reduced SMR, would relate to pre‐HCT FEV_1_. Additionally, we sought to demonstrate the relationship between CT image parameters analysed at chest level (T4) vs. lumbar (L3) level.

## Population and methods

Study population for this analysis included adults ≥18 years of age who had received alloHCT for haematologic malignancies at the H. Lee Moffitt Cancer Center, the single regional site for stem cell transplantation serving the greater Tampa Bay area (pop. 2.7 million). Consecutive patients were included with a diagnosis of lymphoma (March 2005 to April 2015) and all diagnoses (November 2013 to November 2014). Ethical approval was granted by Advarra Institutional Research Board (protocol numbers Pro00015786 and Pro00014686). Eligible subjects required analysable CT imaging of the lumbar or thoracic region within 60 days prior to transplantation. If multiple CT scans were available, CT scan closest to transplant date was analysed. Patient demographics obtained from medical records included age, sex, body mass index (BMI), diagnosis, performance status, previous chemotherapy regimens, length of stay, albumin, and co‐morbidities. Pulmonary function was characterized by FEV_1_ (normalized for age, ethnicity, height, and sex), forced vital capacity (FVC), and the diffusing capacity of the lungs for carbon monoxide (DLCO). FEV_1_ and FVC were captured by spirometry performed within 30 days prior to transplantation, and COPD was defined and graded using FEV_1_ and FVC as per the criteria established by the COPD Foundation.^22^ DLCO was measured by ERS/ATS standards and adjusted for anaemia per guidelines.^23^, ^24^ Co‐morbidities collected were extensive, in accordance with the haematopoietic cell transplantation‐specific co‐morbidity index (HCT‐CI), which is standard for patients who are being considered for alloHCT.^25^ Disease risk index (DRI), developed for the purpose of evaluating patients together across diseases, was utilized to stratify patients according to their level of risk based on diagnosis and staging information.^26^

### Computed tomography assessment

Pre‐transplantation CT images were acquired as part of institutional standard practice and were retrieved from the institutional Picture Archiving Communication System. CT chest (unenhanced) is standard for all haematological malignancies. CT abdomen (contrast‐enhanced) is additionally performed in patients with lymphoma to obtain evidence of remission or disease progression (e.g. review of spleen size, liver, and retroperitoneal lymphadenopathy). Vertebral land‐marking was performed by a board‐certified radiologist. For thoracic series, we identified a single axial CT image landmarked at the fourth thoracic vertebra (T4); for lumbar series, a secondary single axial image at the third lumbar vertebra was selected. Vertebral bodies were selected at the level of the pedicles. Selected images were from the closest date prior to transplant (within 60 days). All images were of 3 mm slice thickness, with a peak kilovoltage of 120 and current varying by patient within the standard algorithms within the SIEMENS CT scanners at this site.

**Figure 1:**
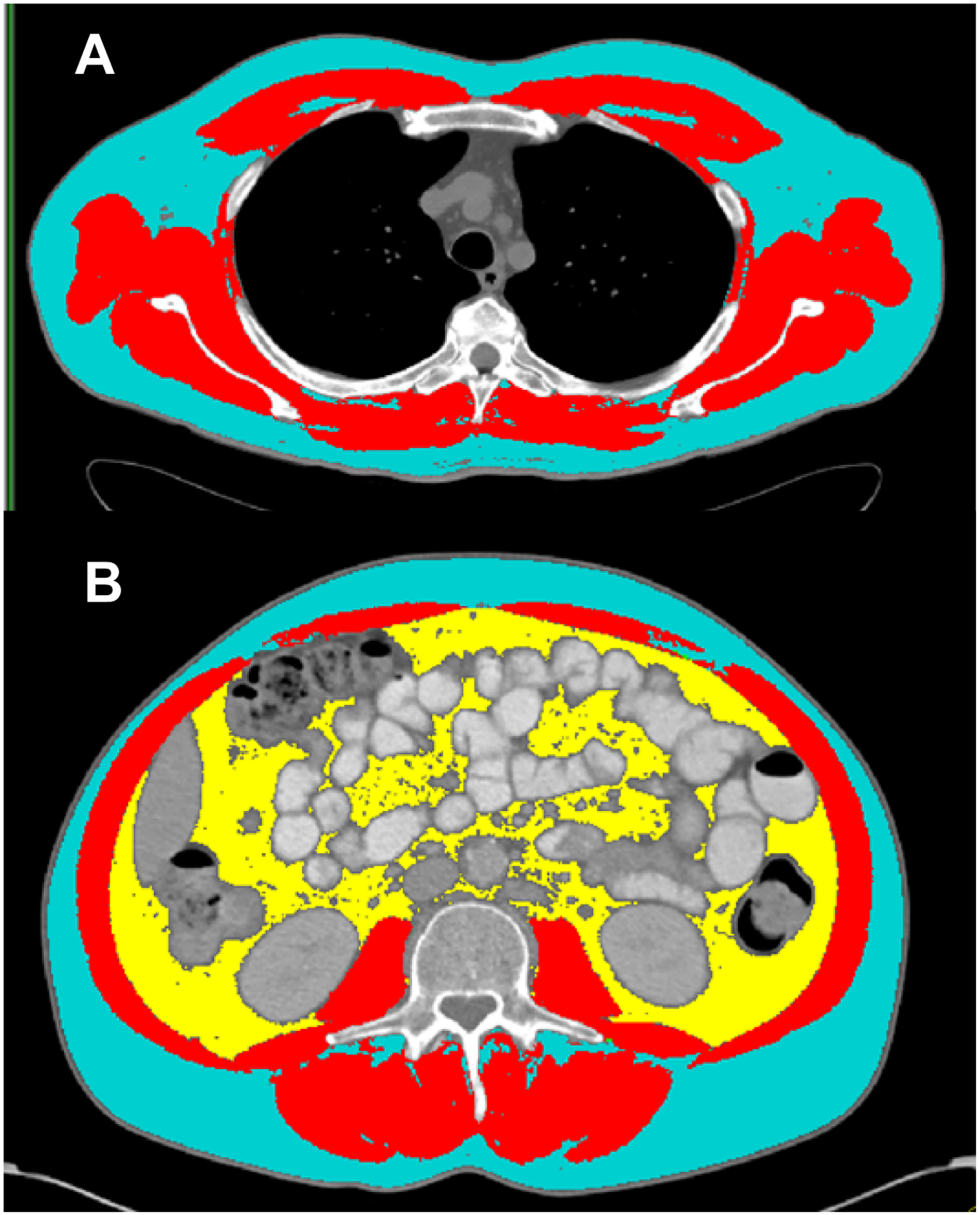
Example thoracic (T4) and lumbar (L3) computed tomography scans with tissue quantification. At the thoracic level (*A*), skeletal muscle is displayed in red, and subcutaneous adipose tissue is displayed in blue. At the lumbar level (*B*), skeletal muscle is displayed in red, subcutaneous adipose tissue in blue, and visceral adipose tissue in yellow.

We quantified tissue cross‐sectional areas with SliceOmatic® (TomoVision, Magog, Quebec, Canada). Predetermined Hounsfield unit (HU) thresholds for muscle were −29 to +150 HU, −50 to −150 HU for visceral adipose tissue (VAT), and −30 to −190 HU for subcutaneous adipose tissue (SAT). Skeletal muscle area (SMA), mean SMR, and areas of VAT and SAT were reported. Adipose tissue outside of the abdominal wall but inside the muscle fascia was included with SAT. Areas of VAT and SAT were summed to obtain the area for total adipose tissue (TAT) at the L3 level (VAT is absent at T4). Example images are present in *Figure* 1 (T4, panel a; L3, panel b). SMA was normalized for height^2^ and reported as SMI (cm^2^/m^2^). For the purpose of comparing the incidence of sarcopenia with reports in the literature, sarcopenia was defined by the widely used lumbar SMI cut‐offs of 52.4 cm^2^/m^2^ for men and 38.5 cm^2^/m^2^ for women.^27^

### Statistics

Frequencies and summary statistics are reported. Comparisons were assessed with parametric tests [independent *t*‐tests,*χ* ^2^ test, or Fisher's exact test (post hoc Bonferroni corrections)]. Correlations were evaluated with Pearson correlation coefficients. To evaluate predictive relationships between our demographic, body composition, and functional variables, we created multivariable linear regression models. For these models, we included both conventional covariates well known to be related to alloHCT outcomes [Karnofsky performance status (KPS), HCT‐CI, and DRI]^26^, ^28^ as well as those related to body composition with previously uncharacterized relationships in this context (SMI and SMR). COPD defined through spirometry was included in predictive models for FEV1 as a co‐morbidity that is generally mild in this population and thus is not comprehensively captured by the HCT‐CI. Ethnicity/race was not evaluated as it has not been shown to impact alloHCT outcomes to this point in US‐based populations.^29^ Analyses were completed using IBM SPSS Statistics for Windows version 23.0 (SPSS, Chicago, IL), and results were considered significant at the *P* < 0.05 level.

## Results

### Demographics

A total of 299 consecutively transplanted patients were identified, of which *n* = 296 (99%) had CT images of acceptable quality within 60 days prior to transplant and were included for this analysis. Patient characteristics are detailed in *Table* 1. There were 161 men (54%) and 135 (46%) women, with an overall mean age of 52.4 ± 12.1 years. Diagnoses spanned indications for alloHCT in haematologic malignancies including non‐Hodgkins lymphoma (*n* = 165), acute myeloid leukaemia (*n* = 66), Hodgkin's disease (*n* = 14), acute lymphocytic leukaemia (*n* = 14), myelodysplastic syndromes (*n* = 18), and other (*n* = 19). Scans were taken a median of 26 (SD 11.5) days prior to stem cell infusion from allogeneic donor, with a majority of scans (69.6%) taken within 30 days of transplantation. Forty‐eight% (*n* = 143) of patients had high co‐morbidity burden (score ≥3) based on pre‐transplant HCT‐CI. Pulmonary decompensation, using defined metrics of COPD,^22^ had a low prevalence—only 10.5% of patients had Stage 1 COPD and 14.5% of patients had Stage U (undefined), as expected in this population. The mean KPS of 90.2 was consistent with ability to carry on normal activity with minor signs or symptoms of the underlying malignancy. The majority of patients (52.7%) were also classified as having an intermediate DRI, whereas the classifications of low risk (22.6%) and high or very high risk (24.7%) were less common. It was also most common that a patient received a myeloablative conditioning regimen (70.6% overall), compared with 29.4% for reduced intensity/non‐myeloablative conditioning. All 296 included subjects had thoracic CT performed during pre‐transplant evaluation capturing the T4 region as is standard in alloHCT. A subset of these (72.6%, *n* = 215) also had an abdominal CT capturing L3. For purposes of consistency with the literature, we performed analyses on the data retrieved from both levels. However for the reason of intrinsic variation of muscle radiodensity at different vertebral levels,^30^ and for the reason that in standard haematological oncology thoracic images are not contrast enhanced but abdominal images are, we have separately analysed thoracic and lumbar regions in all of our analysis.

**Table 1:** Patient demographic characteristics

Characteristic	Overall (*n* = 296)	Male (*n* = 161)	Female (*n* = 135)	*P*‐value
Age (years)	52.4 ± 12.1	52.9 ± 11.5	51.9 ± 12.9	0.477
HCT type				
Allogeneic	296 (100%)	161 (100%)	135 (100%)	
Days CT to transplant	26 (11.5)	27 (13)	25 (11.2)	0.608^c^
Diagnosis, *n* (%)				
Non‐Hodgkins lymphoma	165 (55.7%)	98 (60.9%)	67 (49.6%)	0.394^a^
Acute lymphocytic leukaemia	14 (4.7%)	7 (4.3%)	7 (5.2%)	
Acute myeloid leukaemia	66 (22.3%)	34 (21.1%)	32 (23.7%)	
Hodgkins disease	14 (4.7%)	7 (4.3%)	7 (5.2%)	
Myelodysplastic syndrome	18 (6.1%)	8 (5.0%)	10 (7.4%)	
Other	19 (6.4%)	7 (4.3%)	12 (8.9%)	
HCT‐CI score, *n* (%)				0.597^a^
0	63 (21.3%)	32 (19.9%)	31 (23.0%)	
1	23 (7.8%)	15 (9.3%)	8 (5.9%)	
2	67 (22.6%)	40 (24.8%)	27 (20.0%)	
3	69 (23.3%)	37 (23.0%)	32 (23.7%)	
4+	74 (25.0%)	37 (23.0%)	37 (27.4%)	
Disease risk index, *n* (%)				0.940^a^
Low	67 (22.6%)	36 (22.4%)	31 (23.0%)	
Intermediate	156 (52.7%)	84 (52.2%)	72 (53.3%)	
High/very high	73 (24.7%)	41 (25.5%)	32 (23.7%)	
Regimen intensity				0.798^b^
Myeloablative	209 (70.6%)	115 (71.4%)	94 (69.6%)	
Reduced intensity/non‐myeloablative	87 (29.4%)	46 (28.6%)	41 (30.4%)	
Previous chemotherapy regimens	3.27 ± 1.8	3.28 ± 1.8	3.25 ± 1.9	0.722
KPS	90.2 ± 8.1	90.5 ± 8.0	89.8 ± 8.0	0.447
Albumin	4.08 ± 0.4	4.09 ± 0.4	4.07 ± 0.3	0.702
Prior HCT, *n* (%)	21 (7.1%)	11 (6.8%)	10 (7.4%)	1.0^b^
FEV_1_ (%)	92.0 ± 15.3	89.9 ± 14.8	94.6 ± 15.6	0.009
DLCO	74.5 ± 14.6	76.5 ± 15.6	72.1 ± 12.9	0.009
COPD				0.113^a^
Stage 0 (no COPD)	222 (75.0%)	113 (70.2%)	109 (80.7%)	
Stage 1 (mild)	31 (10.5%)	20 (12.4%)	11 (8.2%)	
Stage 2–3 (moderate–severe)	0 (0.0%)	0 (0.0%)	0 (0.0%)	
Stage U (undefined)	43 (14.5%)	28 (17.4%)	15 (11.1%)	
Lumbar CT Available, n (%)	215 (72.6%)	116 (72.0%)	99 (73.3%)	0.896
Length of Stay	24.0 (6)	23 (5)	24 (6.5)	0.140^c^
T4 body composition				
Skeletal muscle (cm^2^)	183.9 ± 50.7	218.2 ± 40.0	142.9 ± 25.4	<0.001
Subcutaneous adipose tissue (cm^2^)	212.9 ± 103.9	194.9 ± 93.5	234.3 ± 111.7	0.001
Skeletal muscle radiodensity (HU)	38.5 ± 6.8	38.9 ± 7.0	37.9 ± 6.6	0.211
L3 body composition^d^				
Skeletal muscle (cm^2^)	128.2 ± 34.7	152.1 ± 26.7	100.1 ± 18.0	<0.001
Subcutaneous adipose tissue (cm^2^)	198.8 ± 103.6	192.4 ± 102.7	206.2 ± 104.5	0.331
Visceral adipose tissue (cm^2^)	131.3 ± 96.3	170.2 ± 100.3	85.8 ± 67.5	<0.001
Skeletal muscle radiodensity (HU)	35.5 ± 7.3	35.9 ± 7.2	35.2 ± 7.4	0.487
Sarcopenia, *n* (%)	132 (61.4%)	75 (64.7%)	57 (57.6%)	0.326^b^
BMI category, *n* (%)				0.005^a^
Underweight	4 (1.4%)	1 (0.6%)	3 (2.2%)	
Normal weight	89 (30.1%)	36 (22.4%)	53 (39.3%)	
Overweight	116 (39.2%)	68 (42.2%)	48 (35.6%)	
Obese	87 (29.4%)	56 (34.8%)	31 (23.0%)	

Variables are presented as mean ± standard deviation, or median (interquartile range). *P*‐values are Student's *t*‐tests unless otherwise specified. BMI, body mass index; COPD, chronic obstructive pulmonary disease defined by spirometry^22^; CT, computed tomography; DLCO, diffusing capacity of the lung for carbon monoxide, adjusted for haemoglobin; FEV_1_, forced expiratory volume in 1 second; HCT, haematopoietic cell transplantation; HCT‐CI, haematopoietic cell transplantation‐specific co‐morbidity index; HU, Hounsfield unit; KPS, Karnofsky performance status.

^a^ *χ* ^2^ test.

^b^ Fisher's exact test.

^c^ Mann–Whitney *U* test.

^d^ Variables of L3 body composition, including sarcopenia prevalence, have a sample size of 215 (*n* = 99 female, *n* = 116 male).

### Linear regression analysis: FEV_1_

In multivariable linear regression with FEV_1_ as the dependent variable, when we adjusted our model for clinically relevant covariates, age (*P* < 0.0001), sex (*P* = 0.001), HCT‐CI (*P* = 0.010) and COPD grading (*P* < 0.0001), T4 SMI (*P* = 0.023), and T4 SMR (*P* = 0.006) remained as independent predictors, whereas DRI (*P* = 0.877) and KPS (*P* = 0.548) did not (*Table* 2). When evaluating the lumbar region with the same model, age (*P* < 0.0001), sex (*P* = 0.002), HCT‐CI (*P* < 0.0001), COPD (*P* < 0.0001), and L3 SMI (*P* = 0.016) remained as independent predictors. L3 SMR (*P* = 0.106) was no longer an independent predictor, though retained a *β* coefficient (*β* 0.093) only slightly below that of the *β* coefficient (*β* 0.132) founded in the T4 model of greater sample size. KPS (*P* = 0.954) and DRI (*P* = 0.309) were again not associated with FEV1. Conditioning regimen and days between CT transplant both were not associated with FEV1 in all models.

**Table 2:** Linear regression models for prediction of forced expiratory volume in 1 s (FEV_1_) prior to haematopoietic cell transplantation using thoracic (T4) or lumbar (L3) data

Linear Regression Models	*B*	SE *B*	*β*	*t*	*P*‐value
FEV_1_					
Thoracic (*n* = 296)					
Intercept	81.706	9.742		8.387	0.000
Sex (male vs. female)	−5.424	1.639	−0.177	−3.310	0.001
Age	0.204	0.057	0.162	3.594	0.000
Disease risk index	−0.149	0.965	−0.007	−0.155	0.877
HCT‐CI	−0.936	0.360	−0.117	−2.602	0.010
KPS	−0.049	0.082	−0.026	−0.602	0.548
Skeletal muscle index	0.135	0.059	0.127	2.282	0.023
Skeletal muscle radiodensity	0.296	0.106	0.132	2.786	0.006
COPD	−12.010	0.942	−0.573	−12.751	0.000
*R* ^2^	0.492				
Lumbar (*n* = 215)					
Intercept	74.739	11.237		6.651	0.000
Sex (male vs. female)	−6.064	1.928	−0.195	−3.145	0.002
Age	0.285	0.072	0.218	3.950	0.000
Disease risk index	1.191	1.169	0.051	1.019	0.309
HCT‐CI	−1.748	0.431	−0.208	−4.052	0.000
KPS	0.005	0.094	0.003	0.058	0.954
Skeletal muscle index	0.244	0.101	0.153	2.423	0.016
Skeletal muscle radiodensity	0.199	0.123	0.093	1.621	0.106
COPD	−11.989	1.188	−0.536	−10.094	0.000
*R* ^2^	0.516				

COPD, chronic obstructive pulmonary disease, defined by spirometry^22^; HCT‐CI, haematopoietic cell transplantation‐specific co‐morbidity index; KPS, Karnofsky performance status; SE, standard error.

### Linear regression analysis: skeletal muscle index and skeletal muscle radiodensity

For part two of our regression analysis, predicting either SMI or SMR, we created homologous models with sex, age, BMI, DRI, and HCT‐CI as covariates. There were four models—SMI at T4/L3 and SMR at T4/L3 (*Table* 3). For T4 SMI, independent predictors were age (*P* = 0.005), sex (*P* < 0.0001), and BMI (*P* < 0.0001) (*Table* 3). HCT‐CI score (*P* = 0.059) and DRI (*P* = 0.131) were not significant in this model. For L3 SMI, sex (*P* < 0.0001) and BMI (*P* < 0.0001) were independent predictors, while age (*P* = 0.093) fell out of the model. Interestingly, DRI did act as an independent predictor in this instance (*P* = 0.012), while HCT‐CI remained insignificant. At the thoracic level for SMR (T4 SMR), all of our covariates were independent predictors—sex (*P* = 0.009), age (*P* < 0.0001), BMI (*P* < 0.0001), DRI (0.013), and HCT‐CI (*P* = 0.011) significance. At the L3 level, sex (*P* = 0.081) and HCT‐CI (*P* = 0.225) did not achieve significance, while age (*P* < 0.0001), BMI (*P* < 0.0001), and DRI (*P* = 0.019) performed like their counterparts at the T4 level. KPS was not related to either SMI or SMR (data not shown).

**Table 3:** Linear regression models for prediction of pre‐transplant skeletal muscle index and radiodensity using thoracic (T4) or lumbar (L3) data

Linear regression models	*B*	SE *B*	*β*	*t*	*P*‐value
Skeletal muscle index					
Thoracic (*n* = 296)					
Intercept	31.279	4.568		6.847	0.000
Sex (male vs. female)	15.239	1.270	0.530	12.002	0.000
Age	−0.147	0.052	−0.125	−2.842	0.005
BMI	0.707	0.104	0.302	6.821	0.000
Disease risk index	−1.402	0.925	−0.067	−1.514	0.131
HCT‐CI	−0.634	0.334	−0.085	−1.889	0.059
*R* ^2^	0.451				
Lumbar (*n* = 215)					
Intercept	10.762	3.372		3.191	0.002
Sex (male vs. female)	10.103	0.879	0.519	11.491	0.000
Age	−0.062	0.037	−0.076	−1.688	0.093
BMI	0.900	0.086	0.473	10.407	0.000
Disease risk index	−1.655	0.656	−0.114	−2.522	0.012
HCT‐CI	−0.229	0.240	−0.044	−0.955	0.341
*R* ^2^	0.585				
Skeletal muscle radiodensity					
Thoracic (*n* = 296)					
Intercept	58.578	2.533		23.124	0.000
Sex (male vs. female)	1.838	0.704	0.135	2.611	0.009
Age	−0.174	0.029	−0.310	−6.043	0.000
BMI	−0.360	0.057	−0.324	−6.272	0.000
Disease risk index	−1.281	0.513	−0.129	−2.495	0.013
HCT‐CI	−0.473	0.185	−0.133	−2.559	0.011
*R* ^2^	0.252				
Lumbar (*n* = 215)					
Intercept	59.719	3.340		17.880	0.000
Sex (male vs. female)	1.529	0.871	0.105	1.756	0.081
Age	−0.253	0.036	−0.411	−6.933	0.000
BMI	−0.347	0.086	−0.243	−4.050	0.000
Disease risk index	−1.541	0.650	−0.142	−2.370	0.019
HCT‐CI	−0.289	0.238	−0.073	−1.216	0.225
*R* ^2^	0.274				

BMI, body mass index; HCT‐CI, haematopoietic cell transplantation‐specific co‐morbidity index; SE, standard error.

### Sarcopenia prevalence

Weight and body composition features are provided in *Table* 1. Mean BMI was high for both men and women, demonstrating an overweight population—69% of subjects were overweight (BMI 25–29.9) or obese (BMI 30.0+). The majority of men and women met criteria for sarcopenia (♂64.7%; ♀57.6%) based on published cut‐offs at the L3 level.^27^ These overall rates of sarcopenia were markedly higher (*P* < 0.001) in alloHCT recipients than patients with locally advanced or metastatic malignancies of the lung or gastrointestinal tract (♂56.6%; ♀36.0%).^31^ As seen in *Table* 4, the difference in sarcopenia prevalence is most notable when comparing across age‐matched and sex‐matched cohorts. When comparing with those with solid tumours, alloHCT patients in our study had a significantly higher prevalence of sarcopenia. This included men (*P* = 0.012) and women (*P* = 0.014) aged 50–60 and was even more prominent in men (*P* = 0.004) and women (*P* = 0.002) aged 60–70. These rates of sarcopenia founded in alloHCT patients were systematically higher than expected for their chronological age; for example, male alloHCT recipients aged 50–60 years were found to have similar rates of sarcopenia (74.4%) as seen in men 20 years their senior (70–80) with metastatic solid tumours. As another point of reference, we have included a population of healthy community‐dwelling young adults in *Table* 4 (from reference 30).

**Table 4:** Comparison of sarcopenia prevalence in patients having haematopoietic cell transplantation versus advanced solid tumour patients (from reference^31^), versus healthy young adults (from reference^30^)

Population	*n*	SMI	Sarcopenia prevalence (%)	*P*‐value
**Healthy young**				
Male mean age = 30.9 y	317	60.9 ± 7.8	n/a	
Female mean age = 31.2 y	410	47.5 ± 6.6	n/a	
**Cancer**				
Age group: 50–60 y				
**Solid tumour**				
Male	178	52.6 ± 8.4	52.2	
Female	135	41.9 ± 6.6	34.1	
**alloHCT**				
Male	39	48.7 ± 7.4	74.4	0.012^*^
Female	37	37.8 ± 5.4	56.8	0.014^*^
Age group: 60–70 y				
**Solid tumour**				
Male	260	52.6 ± 9.6	48.1	
Female	200	41.9 ± 7.3	32.0	
**alloHCT**				
Male	35	46.6 ± 9.1	74.3	0.004^*^
Female	30	37.0 ± 8.6	63.3	0.002^*^

Prevalence of sarcopenia according to the criteria of Prado *et al*.^27^ alloHCT, allogeneic haematopoietic stem cell transplant; n/a, not applicable; SMI, skeletal muscle index.

^*^ *P*‐values calculated by Fisher's exact test, between the age‐matched and sex‐matched tumour groups.

Sarcopenia prevalence is not easily compared with data in the literature as different methods are used to quantify muscle and different sarcopenia thresholds are used; however, prevalence of sarcopenia in community‐dwelling American men ≥80 years of age is about 50%,^32^ much lower than our findings in alloHCT patients.

### Fourth thoracic vs. third lumbar comparison

**Figure 2:**
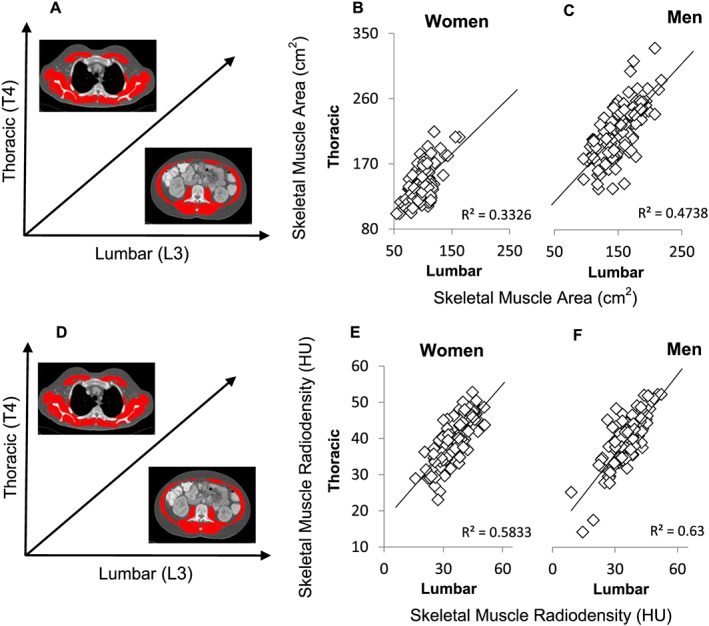
T4 vs. L3 correlations for muscle characteristics (skeletal muscle area/skeletal muscle radiodensity). Panel (*A*) illustrates the vertebral landmark and tissue on the axes of panels (*B*) and (*C*); panel (*D*) does the same for (*E*) and (*F*). Correlations were assessed between skeletal muscle area and skeletal muscle radiodensity at two vertebral levels and are presented for male (*n* = 116; panels *C*/*F*, respectively) and female (*n* = 99; panels *B*/*E*, respectively) patients. HU, Hounsfield unit.

**Figure 3:**
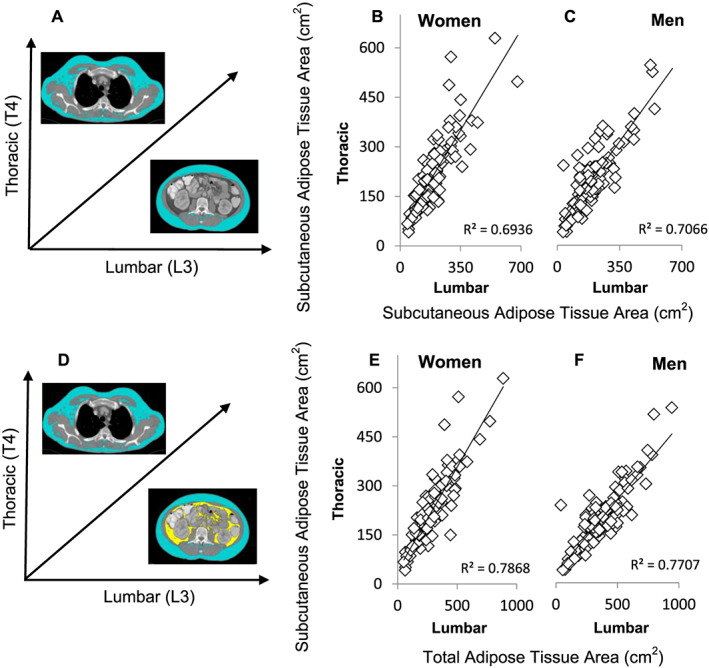
T4 vs. L3 correlations for characteristics of adiposity (subcutaneous adipose tissue/total adipose tissue). Panel (*A*) illustrates the vertebral landmark and tissue on the axes of panels (*B*) and (*C*); panel (*D*) does the same for (*E*) and (*F*). Correlations were assessed between subcutaneous adipose tissue at two vertebral levels and of T4 subcutaneous adipose tissue vs. total adipose tissue at the L3 level, which consists additionally of visceral adipose tissue. Values are presented for male (*n* = 116; panels *C*/*F*, respectively) and female (*n* = 99; panels *B*/*E*, respectively) patients.

When comparing T4 and L3 regions of the body within patients, men and women were both more muscular at T4 than at L3. Correlative analyses performed for T4 vs. L3 demonstrated moderate correlation for skeletal muscle parameters SMA (women: *r* ^2^ = 0.33, *P* < 0.001; men: *r* ^2^ = 0.47, *P* < 0.001, *Figure* 2B and 2C) and SMR (women: *r* ^2^ = 0.58, *P* < 0.001; men: *r* ^2^ = 0.63, *P* < 0.001, *Figure* 2E and 2F). Subcutaneous adipose tissues at the T4 vs. L3 level had higher degrees of correlation, with women having an *r* ^2^ value of 0.69 (*P* < 0.001) and men having a value of 0.71 (*P* < 0.001) (*Figure* 3B and 3C). Lastly, the highest correlation coefficients we observed were in comparing TAT at the L3 level (consisting of SAT + VAT) with SAT at the T4 level (which is considered TAT, as the only adipose tissue constituent present at this level), an *r* ^2^ value of 0.79 for women (*P* < 0.001) and 0.77 for men (*P* < 0.001) (*Figure* 3E and 3F).

## Discussion

Herein, we describe a population‐based cohort of allogeneic HCT recipients evaluated for sarcopenia. Of the many techniques currently available to assess both muscle mass and adipose tissue, CT scan analysis allows for precise and specific examination of SMI and radiodensity and is considered to be ‘gold standard’ for estimating sarcopenia.^33^, ^34^ This report displays the feasibility of evaluating body composition via CT imaging in an HCT recipient cohort, given the frequency of CT scans performed for disease assessment or infection monitoring purposes, as is performed routinely during transplant candidacy evaluation. This approach has been adopted in recent studies^35^, ^36^; however, pulmonary function was not a focus of those works. Moreover, we demonstrate the high prevalence of sarcopenia amongst alloHCT recipients, with the majority of recipients having evidence of sarcopenia prior to HCT; although not studied here, it may be expected that the extremely high dose chemotherapy used to ablate the bone marrow would induce even further large magnitude muscle loss.^14^, ^37^ Notably, while sarcopenia was identified in the majority of our cohort, currently utilized traditional measurements of performance status (KPS) did not predict deficits in muscle mass, radiodensity, or FEV_1_ within this group. The mean KPS in our cohort was 90.2 ± 8.1, consistent with ability to carry on normal activity, and only 4.1% of patients had KPS <80. Our results suggest that KPS, as assessed by the medical team, has limited association with muscle mass and may need to be supplemented by sarcopenia measurement.

The importance of assessment of SMI and radiodensity in HCT recipients has been previously underscored by physical functional decline after transplantation as assessed by the Medical Outcomes Study Short‐Form 36 questionnaire^38^, ^39^ with full physical recovery often requiring several years after allograft^38^, ^40^, ^41^ and predisposing patients to ongoing medical risk.^41^ Our report suggests that a high proportion of alloHCT recipients could potentially be predisposed to sequelae of sarcopenia such as transplant‐related morbidity or mortality that would otherwise not be identified as such without CT quantification of the characteristics of their musculature. An investigation of mortality in our population will be the subject of a future report. The high prevalence of sarcopenia in this curative intent population is particularly striking when comparing across age‐matched and sex‐matched solid tumour patients receiving palliative chemotherapy.^31^ Additionally, the mean age in our cohort was 52.4 ± 12.1; the degree of sarcopenia in this population was comparable with solid tumour patients approximately 20 years their senior and above that of community‐dwelling seniors in their 80s.

Current transplant physician attitude towards patient tolerance of alloHCT procedure is influenced by the assessment of co‐morbidity burden, performance status, and chronological age^1^ due to their association with inferior outcomes.^25^, ^28^, ^42^, ^43^, ^44^ However, assessment of ‘biological’ or ‘functional’ age vs. chronological age remains a challenge. Our study demonstrates the association of SMI and SMR with FEV_1_ by pulmonary function testing, an objective measurement associated with functionality, mortality, and potential long‐term morbidity in allograft recipients.^14^, ^15^, ^16^, ^17^, ^25^, ^44^, ^45^ Age and sex are known factors associated with FEV_1_ as also seen in our cohort; however, both skeletal muscle quantity and fatty infiltration as measured by SMI and SMR, respectively, are independent predictors of FEV_1_ even when the models are heavily adjusted for clinical covariables including mild intrinsic pulmonary disease (COPD). Pulmonary involvement by the cancer itself is rarely seen in either AML or MDS^46^ as they are diseases of the bone marrow micro‐environment, and while the other diseases could have pulmonary involvement, the vast majority of patients are in complete remission at time of transplant and do not have any discernible disease in the lungs. Acceptable pulmonary function is an eligibility criterion for transplantation as this treatment entails risk of pulmonary complications, so the FEV_1_ is uniformly high; however, this does not necessarily indicate ideal pulmonary function as some transplant eligible patients in our population had low grade pulmonary decompensation. Inconsistency of FEV_1_ and pulmonary symptoms has also been reported, and patients with preserved pulmonary function remain at risk for pulmonary complication and activity limitations.^47^

Body composition evaluation can be assessed in both the thoracic and lumbar regions; here, we present the first study in alloHCT that includes full analysis at both vertebral levels. This is significant when considering sarcopenia evaluations, and only a certain radiographic field is available; our analyses demonstrate that many of the same results can be produced by data retrieved from T4 vs. L3, with some variation. While there are differences in quantitative amounts of skeletal muscle and adipose mass as expected between chest and abdomen, they are however correlated. Transplant‐related factors are predictive of SMI in both the chest and abdomen. Interestingly, in both the thoracic and abdominal regions, HCT‐CI was not associated with sarcopenia demonstrating the independent value of body composition not otherwise captured in a co‐morbidity evaluation.

### Limitations

This study had the inherent limitations of a retrospective design. Future studies are required to look at the association between SMI/SMR and morbidity/mortality post‐transplantation.

In summary, body composition assessments in alloHCT recipients are feasible. Similar conclusions based on thoracic and lumbar CT imaging help to broaden the evaluable patient population, as use can be made of thoracic or lumbar regions. Evaluation of sarcopenia may further be valuable in risk‐stratifying patients beyond currently known paradigms and may be a composite evaluation of biological age including actual years, co‐morbidities, sex, and body mass.

## Conflict of interest

A.M., K.D.B., M.E., R.F., K.T., J.A.P., and V.E. B. declare that they have no conflict of interest.
